# Practical Dietetics with Reference to Diet in Disease

**Published:** 1905-06

**Authors:** 


Practical Dietetics with Reference to Diet in Disease. By Alida Frances Pattee, Instructor in Dietetics Bellevue Training School for Nurses, Bellevue Hospital, New York City. Second edition. Duodecimo, pp. 311. Published by the Author, 52 West Thirty-ninth Street, New York City. (Price, $1.00.)

This book is the outgrowth of the author's work as an instructor in dietetics in various hospitals. It contains recipes for the preparation of various invalid foods, a number of well-selected diet lists, and several formulæ for the preparation of infant foods. Except in the latter particular it is very satisfactory. Nurses ought to find it a valuable addition to their libraries. M. J. F.

